# Anxiolytic-like effects observed in rats exposed to the elevated zero-maze following treatment with 5-HT_2_/5-HT_3_/5-HT_4_ ligands

**DOI:** 10.1038/srep03881

**Published:** 2014-01-24

**Authors:** Rob Bell, Aaron A. Duke, Paula E. Gilmore, Deaglan Page, Laurent Bègue

**Affiliations:** 1 School of Psychology, Queen's University, Belfast, Northern Ireland, UK; 2 Department of Psychology, University of Kentucky, USA; 3 Department of Psychology, Grenoble-Alpes University, France

## Abstract

The present study examined the effects of administering selective 5-HT antagonists and agonists to rats tested in the elevated zero-maze (EZM) model of anxiety. The EZM paradigm has advantages over the elevated plus-maze (EPM) paradigm with respect to measuring anxiety, yet has been utilized less frequently. Three experiments were conducted each with a diazepam control (0.25, 0.5 and 0.75 mg/kg). In the first experiment, we administered the 5-HT_2C_ antagonist RS 102221 (0.5, 1.0, and 2.0 mg/kg) and 5-HT_2C_ agonist MK-212 (0.25, 0.5 and 0.75 mg/kg); in the second experiment, we administered the 5-HT_3_ antagonist Y-25130 (0.1, 1.0 and 3.0 mg/kg) and 5-HT_3_ agonist SR 57227A (0.1, 1.0 and 3.0 mg/kg), and in the third experiment, we administered the 5-HT_4_ antagonist RS 39604 (0.01, 0.1, 1.0 mg/kg) and 5-HT_4_ agonist RS 67333 (0.01, 0.1 and 0.5 mg/kg). The administration of 5-HT_2/3/4_ subtype antagonists all generated behavioral profiles indicative of anxiolytic-like effects in the EZM, which was apparent from examination of both traditional and ethological measures. While little effect was observed from 5-HT_2_ and 5-HT_3_ agonists, the 5-HT_4_ agonist RS 67333 was found to produce a paradoxical anxiolytic-like effect similar to that produced by the 5-HT_4_ antagonist RS 39604. We conclude by discussing the implications of these findings.

Interest in the role of the 5-HT receptors in the control of anxiety first arose when clinical trials revealed an anxiolytic-like effect of the non-selective 5-HT_2_ antagonist ritanserin in humans^1^. Since this time a number of preclinical trials have been carried out, the majority of which have employed non-selective ligands. However, an increasing awareness of the complexity of the 5-HT receptor family^2^ coupled with a number of equivocal findings for non-selective 5-HT agents has led to great importance being placed upon the need to test the role of specific 5-HT receptor subtypes in regulating anxiety.

Although preliminary studies employing animal models of anxiety have, in general, demonstrated that a reduction in 5-HT neurotransmission plays a role in the effects of novel non-benzodiazepine anxiolytics^3^, equivocal results for the effects of 5-HT subreceptor ligands have been reported, particularly in studies using the elevated plus-maze paradigm (EPM)^4^. Inconsistent behavioral profiling of drugs that modulate the 5-HT system led earlier investigators to query the utility of the EPM paradigm^5^; specifically, the predictive validity of the EPM appears to be limited to benzodiazepine-related compounds^6^.

The elevated zero-maze (EZM) paradigm represents an important improvement over the EPM in that it removes any ambiguity in the interpretation of time spent on the hub of the EPM and permits continuous exploration^7^,^8^,^9^. The present paper will compare data obtained in our laboratory using 5-HT_2_, 5-HT_3_ and 5-HT_4_ receptor ligands tested using the EZM paradigm with the results from other studies that have utilized similar receptor ligands in a variety of preclinical models of anxiety. Specifically, we tested the effects of the 5-HT_2C_ antagonist RS-102221, the 5-HT_2C_ agonist MK-212, the 5-HT_3_ antagonist Y-25130, the novel 5-HT_3_ agonist SR 57227A, the selective 5-HT_4_ antagonist RS 39604, and the high-affinity 5-HT_4_ partial agonist RS 67333.

The roles of 5-HT_2_^3^, 5-HT_3_^10^ and 5-HT_4_ receptors^11^ have been extensively reviewed in a variety of animal models of anxiety. In addition, comprehensive reviews^4^,^12^,^13^,^14^ have examined, inter alia, strategies for experimental modeling of anxiety, the validity of rodent models of anxiety and discussed the progression of such paradigms.

## Results

Combined descriptive statistics for the study variables including time spent on open arms, open arm entries, head dips, SAP, risk assessment, and rearing duration/frequency are presented in Table 1 along with the correlations between aggregated study variables. Tables 2,3,4 respectively include the specific results for the three experiments. Finally, Table 5 includes a summary of the direction of statistically significant findings across all three experiments.

### Experiment 1: 5-HT_2_ receptor ligands

Analyses revealed significant variation in SAP, head dips, and risk assessment behaviors across the various doses of diazepam. Conversely, diazepam did not lead to significant variation in time spent on the open arms of the maze, number of entries into the open arms, and duration and frequency of rearing behaviors. Follow-up Mann-Whitney *U* tests revealed an increased number of head dips for the highest two doses (*U* = 22.0, *p* < 0.05; *U* = 2.0, *p* < 0.01). All three doses of diazepam were associated with reductions in SAP (*U* = 8.5, *p* < 0.001; *U* = 16.0, *p* < 0.01; *U* = 5.5, *p* < 0.001) and risk assessment (*U* = 13.0, *p* < 0.01; *U* = 18.0, *p* < 0.05; *U* = 13.0, *p* < 0.01).

RS 102221 dose conditions produced significant variation with respect to time spent on open arms, number of open arm entries, SAP, head dips, and risk assessment. Follow-up post-hoc analyses revealed that all doses of RS 102221 were associated with reduced SAP (*U* = 9.5, *p* < 0.01; *U* = 3.0, *p* < 0.001; *U* = 2.0, *p* < 0.001) while only the highest dose of 2.0 mg/kg was associated with increased time on the open arms (*U* = 22.5, *p* < 0.05), and head dips (*U* = 10, *p* < 0.01). Analyses revealed a significant reduction in risk assessment at both the 1.0 mg/kg (*U* = 17.0, *p* < 0.05) and 2.0 mg/kg (*U* = 13.0, *p* < 0.01) doses.

Significant variation was observed across MK-212 dose conditions for rearing duration and rearing frequency. All doses were linked to decreased rearing duration (*U* = 20.0, *p* < 0.05; *U* = 23.0, *p* < 0.05; *U* = 5.0, *p* < 0.001) with the 0.25 mg/kg and 0.75 mg/kg doses being also linked to decreased rearing frequency (*U* = 15.0, *p* < 0.01; *U* = 2.0, *p* < 0.001).

### Experiment 2: 5-HT_3_ receptor ligands

Analyses revealed that the highest dose of diazepam (0.75 mg/kg) significantly decreased the duration of risk assessment behavior (*U* = 16.0, *p* < 0.01). The highest two doses of diazepam (0.5 mg/kg and 0.75 mg/kg) were associated with decreased SAP (*U* = 22.0, *p* < 0.05; *U* = 17.5, *p* < 0.05) and the 0.5 mg/kg dose was also associated with increased head dips (*U* = 23.5, *p* < 0.05).

While no significant differences were observed for the 0.1 mg/kg and 3.0 mg/kg doses of Y-25130, a number of effects were observed for the moderate 1.0 mg/kg dose. Specifically, the 1.0 mg/kg Y-25130 dose was associated with significantly increased time spent in the open arms (*U* = 17.0, *p* < 0.05) and a greater number of open arm entries (*U* = 17.5, *p* < 0.05). Additionally, this dose was associated with decreased SAP (*U* = 23.0, *p* < 0.05), decreased risk assessment (*U* = 16.0, *p* < 0.01), and increased head dips (*U* = 23.0, *p* < 0.05).

Mann-Whitney *U* analysis revealed that the moderate dose of SR 57227A (1.0 mg/kg) was associated with a significant increase in risk assessment (*U* = 16.0, *p* < 0.01). No other statistically significant effects were observed across the 0.1 mg/kg and 3.0 mg/kg doses of SR 57227A.

### Experiment 3: 5-HT4 receptor ligands

Post-hoc analysis revealed that both the 0.5 and 0.75 mg/kg doses of diazepam significantly increased time spent on open arms (*U* = 11.0, *p* < 0.01; *U* = 13.0, *p* < 0.01), number of open arm entries (*U* = 23.5, *p* < 0.001; *U* = 23.5, *p* < 0.05), and number of head dips (*U* = 9.0, *p* < 0.01; *U* = 19.0, *p* < 0.05), while significantly decreasing duration of risk assessment behavior (*U* = 8.0, *p* < 0.01; *U* = 18.0, *p* < 0.05). Additionally, the 0.75 mg/kg dose was linked to decreased rearing duration (*U* = 21.5, *p* < 0.05).

Mann-Whitney *U* analysis revealed that all doses of RS 39604 were associated with significant increases in time spent on open arms (*U* = 1.0, *p* < 0.01; *U* = 8.0, *p* < 0.01; *U* = 6.0, *p* < 0.01) an increased number of open arm entries (*U* = 14.3, *p* < 0.01; *U* = 15.0, *p* < 0.01; *U* = 6.0, *p* < 0.01), increased head dips (*U* = 5.0, *p* < 0.01; *U* = 13.5, *p* < 0.01; *U* = 12.5, *p* < 0.01), and significantly decreased risk assessment (*U* = 3.0, *p* < 0.01; *U* = 5.0, *p* < 0.01; *U* = 5.0, *p* < 0.01).

Analyses revealed that all doses of RS 67333 were associated with an increased number of open arm entries (*U* = 9.0, *p* < 0.01; *U* = 23.0, *p* < 0.05; *U* = 23.5, *p* < 0.05) and decreased risk assessment duration (*U* = 3.0, *p* < 0.01; *U* = 6.0, *p* < 0.01; *U* = 20.5, *p* < 0.01). Furthermore, both the 0.01 mg/kg and 0.1 mg/kg doses of RS 67333 were linked to increased head dips (*U* = 10.5, *p* < 0.01; *U* = 12.0, *p* < 0.01) and time spent on the open arms of the maze (*U* = 13.0, *p* < 0.01; *U* = 11.0, *p* < 0.01).

## Discussion

While both the 0.5 and 1.0 mg/kg doses of the 5-HT_2C_ antagonist RS 102221 failed to significantly modify spatio-temporal measures in the current experiment, the 2.0 mg/kg dose of this drug significantly increased time spent on the open arms of the EZM. This pattern of effects suggests a possible anxiolytic role for higher doses of RS 102221. Consistent with this interpretation, an ethological measure indicative of reduced anxiety (i.e., head dips) was significantly increased only at the 2.0 mg/kg dose. While one earlier experiment failed to find any anxiolytic effects for the 5-HT_2A/2C_ antagonist ketanserin^3^, our outcome is consistent with an earlier report of an anxiolytic-like effect of the 5-HT_2B/2C_ antagonist SB 200646A^27^ and other reports of anxiolytic-like action of 5-HT_2C_ blockade in animal models of anxiety^28^.

An examination of traditional measures of anxiety revealed a general lack of effect for the selective 5-HT_2C_ agonist MK-212. However, MK-212 was found to significantly reduce the duration of rearing at all doses and to significantly reduce the frequency of rearing at both the lowest and highest doses tested. Factor analysis has found rearing to load on a ‘motor activity' factor in the EPM^29^ suggesting the possibility that the general lack of effects observed after MK-212 administration may have been due to behavioral inhibition/sedation. Overall, these findings are in agreement with a number of previous studies which also report a general lack of effect of 5-HT_2_ agonism in animal models of anxiety^3^,^30^,^31^.

The finding that the 1.0 mg/kg dose of Y-25130 increased both time spent on the open arms and total arm entries is in agreement with previous studies examining the effects of 5-HT_3_ antagonism on traditional measures in both the EPM and EZM^32^,^33^ and is compatible with an anxiolytic-like profile comparable to that obtained with diazepam. Consistent evidence for this interpretation was also observed from ethological measures with the 1.0 mg/kg dose leading to decreased SAP and risk assessment in conjunction with increased head dips. This latter finding is also in agreement with previous studies including work with the EPM^34^, social interaction test^35^, and light-dark test^17^, in suggesting an anxiolytic effect of 5-HT_3_ antagonists in animals. However, it should be noted that these effects were not observed for the smaller (0.1 mg/kg) and larger (3.0 mg/kg) doses suggesting the possibility of a hormetic dose-response curve.

While the 5-HT_3_ agonist SR 57227A was found to significantly modify most anxiety-related behaviors as measured in the EZM, examination of the effects of specific doses revealed a general lack of significant effects when compared to the saline control condition. The 0.1 mg/kg dose of this drug failed to modify any behavioral measures, although the general pattern of results, which include non-significant increases in behavior associated with both anxiogenesis and anxiolysis, would suggest non-specific effects.

The 5-HT_4_ antagonist RS 39604 significantly increased both time spent on the open arms and total arm entries in a manner consistent with anxiolysis. These findings are in agreement with those of Silvestre and colleagues^11^ as well as Kennett and colleagues^28^,^36^ who previously reported an anxiolytic-like effect of the 5-HT_4_ antagonists GR 113808, SB 204070, SB 204070A, and SB 207266A on traditional measures in the EPM. With respect to ethological measures, RS 39604 was found to increase head dips and reduce risk assessment, but failed to modify SAP. Overall, RS 39604 was found to modify less anxiety-related behaviors than diazepam in the current experiment. This finding is again in agreement with the work of Silvestre et al.^11^ and Kennett et al.^28^,^36^, both of whom reported that the effects of 5-HT_4_ antagonists were smaller than those of either diazepam or chlordiazepoxide in the EPM, leading both authors to conclude that 5-HT_4_ antagonists have modest anxiolytic-like activity when compared with the benzodiazepines.

The 5-HT_4_ agonist RS 67333 was found to increase both time on the open arm of the EZM and to increase the number of entries onto open arms, a profile consistent with anxiolysis. This interpretation is further supported by ethological measures (i.e., all doses resulted in a reduction in risk assessment with the 0.01 mg/kg and 0.1 mg/kg doses were associated with an increase in head dips). While this finding is in agreement with previous findings in suggesting that 5-HT_4_ agonism is associated with a decrease in anxiety-induced behavioral inhibition^37^, the result is surprising in light of the finding that 5-HT_4_ *antagonism* led to a similar anxiolytic profile. One possible explanation for this seeming paradox is a non-rectilinear relation between 5-HT_4_ activity and anxiety. An inverted-U shaped anxiolytic profile would be sensitive to 5-HT_4_ disruptions whether they were in the form of increased or decreased 5-HT_4_ activation. However, further research will be necessary to develop this possibility.

In conclusion, results from this study indicate a potential role for 5-HT_2_, and 5-HT_3_ antagonists and an apparently paradoxical role for both 5-HT_4_ agonists and 5-HT_4_ antagonists in producing anxiolytic-like effects on rats tested in the EZM paradigm. Such results from the use of the EZM can be compared to data from the EPM that is considered comparable following diazepam challenge, although the more direct measure of time spent in the open may represent an advantage of the elevated zero-maze^7^.

The results for the 5-HT_2_, 5-HT_3_ and 5-HT_4_ antagonists employed in our experiments are consistent with the original hypothesis for the role of serotonin in the pathogenesis of anxiety that was predicated on an association between a reduction in 5-HT turnover and the anxiolytic effects of benzodiazepines suggesting that a reduction of 5-HT neurotransmission results in an anxiolytic-like effect, whereas increased activity produces an anxiogenic-like effect^38^. Notwithstanding this conclusion, the behavioral pharmacology of 5-HT receptor ligands is often more inconsistent than the effects of standard anxiolytics and not all results are explainable in terms of the classic hypothesis, as evinced in our third experiment that demonstrated an anxiolytic-like effect with a compound that increased serotonergic activity.

Moreover, it should be noted standard anxiolytics (e.g., diazepam) sometimes produce counter-intuitive results as well such as the finding in Experiment 2 (see Table 3) that the low dose of diazepam was associated with less time spent on the open arms of the EZM than the saline control condition. Given that data for these groups were collected over a time span of 18 months, we postulated that such variations may, in part, be due to circadian rhythm effects. Benzodiazepines, such as diazepam, are thought to act by potentiating the action of the neurotransmitter γ-aminobutyric acid, which in turn, has been linked to regulation of the sleep-wake cycle^39^. Hence, the variations in responses to diazepam may be the result of the benzodiazepine acting at varying levels of GABA that mediate the generation of circadian rhythms.

A dual role for 5-HT in the mediation of different types of fear has been posited by Graeff and Zangrossi^40^. Specifically, serotonin may either enhance or reduce anxiety-like behavior depending upon the receptor subtype involved. Indeed, the fact that selective serotonin reuptake inhibitors are efficacious in the treatment of generalized anxiety disorder and in panic disorder indicates that there are conditions in which increased 5-HT activity can reduce anxiety.

## Methods

### Animals

All work was licensed by the Home Office via the Northern Ireland Department of Health, Social Services and Public Safety in accordance with the UK Animals (Scientific Procedures) Act 1986 and was in full compliance with Queen's University Belfast's Policy on the Use of Animals in Research and Teaching. In each of the three experiments, one hundred male Sprague-Dawley rats were randomly assigned as follows: saline control (*n* = 10), diazepam (*n* = 30), 5-HT antagonist (*n* = 30), and 5-HT agonist (*n* = 30). Animals were supplied from a breeding stock at Queen's University and weighing between 280 and 350 grams were group-housed (5 per cage; cage size 51 × 39 × 19 cm) in a temperature-controlled environment 22 ± 1°C under a 12-hour reverse light-dark cycle (lights off at 0800 hours) for four weeks prior to testing and prior to experimentation has been handled only for routine husbandry. Food and water were available ad libitum. All animals were both drug and experimentally naive.

### Apparatus

The design of the maze employed was based on that originally proposed by Shepherd et al.^9^ and consists of a black Perspex annular platform, 105 cm in diameter and 10 cm wide, elevated 65 cm above the ground. The maze was divided into four equal quadrants, two of which were enclosed with black Perspex walls, 27 cm in height. The walls were on both the inner and outer sides of the platform so as to provide the ‘closed' areas of the maze. The ‘open' arms had no walls, but did have a 1 cm lip which acts as a tactile aid to animals when on the open areas of the maze. Lighting was provided by two 60 watt red ceiling lights at a height of 2 m positioned directly over the maze and one 60 watt red bulb positioned closer to the maze. Two cameras poised at 45-degree angles were positioned 1 m above the ground and 1 m from either side of the maze. The cameras were connected to a multiplexer in an adjacent laboratory in order to prevent distractions and provided continuous recording of behavior for subsequent analysis.

### Drugs

All of the active compounds–MK-212 (0.25–0.75 mg/kg), RS 102221 (0.5–2.0 mg/kg), SR 57227A (0.1–3.0 mg/kg), Y-25130 (0.1–3.0 mg/kg), RS 67333 (0.01–0.5 mg/kg), RS 39604 (0.01–1.0 mg/kg), and the positive comparator, diazepam (0.25–0.75 mg/kg), were obtained from Tocrais Cookson UK. The dose ranges for the drugs were chosen from the experimental pharmacology literature examining the relation between 5-HT and anxiety^3^,^4^,^15^,^16^,^17^,^18^,^19^,^20^. All compounds were prepared as solutions or suspensions in physiological vehicle. Saline alone was administered to control animals and diazepam was used as a positive control. All solutions were freshly prepared on the day of testing and administered intraperitoneally (1 ml/kg) 30 minutes prior to testing.

### Procedure

All testing was conducted between 0800 and 1400 hours. On the day of testing one animal was selected and transferred in a covered box (to maintain darkness) measuring 32 × 16 × 12.5 cm to a room adjacent to the experimental room. Here a subcutaneous (s.c) injection was administered under red light and the animal was placed in a cage measuring 40.5 × 28 × 12 cm containing only sawdust bedding (i.e., no food and water). The animal remained in this staging cage for 30 minutes in order to allow adequate absorption of drug, after which time the animal was transferred to the experimental room (again in a darkened box) and placed on the zero-maze at a junction between an open and closed arm, facing into the closed area. Each animal was placed on an alternative side of the maze so as to avoid any bias. All testing was conducted under red-light. After 5 minutes, the animal was removed from the maze and the maze was washed using an ethanol/water solution so as to avoid olfactory cues transferring from one experimental session to the next. Once testing was complete, computer-based event recording and ethological analysis software (Hindsight ver. 1.5) was used to register the relevant experimental variables. Videotapes were scored blindly by a highly trained observer (intra-observer reliability > 0.8). Behavioral parameters comprised traditional spatio-temporal, locomotion, maintenance and ethological measures^21^,^22^,^23^, which are outlined below.

### Measures

#### Spatio-temporal measures

Traditional spatio-temporal measures included (a) time on open arms and (b) frequency of open arm entries. The animal was judged to enter into the open arm area of the maze when all four paws crossed the open arm threshold. After crossing into the open arm area, the animal was allowed to have one paw in the closed area of the maze and still be considered in the open area; however, if two paws crossed the threshold, then they were judged to have re-entered the closed area of the maze^9^,^24^.

#### Ethological measures

*Stretched Attend Posture* was defined as the frequency with which the animal makes a forward elongation of the head and shoulders followed by a retraction to the original position when the animal is on an open arm^23^. *Head dips* measured the frequency of the animal protruding its head over the ledge of an open arm and down towards the floor^25^. Finally, *Risk Assessment* was scored as the frequency of an animal exiting a closed arm of the maze with forepaws and head only, and investigating the surroundings. Risk assessment was scored independently of stretch attend posture, which often accompanied risk assessment^21^.

#### Locomotion and maintenance measures

*Rearing Frequency* was defined as the number of vertical movements against the side or end of walls of the closed arms of the maze or raising up its hind paws sans support in the open area of the maze^26^. *Rearing Duration* measured the time each animal was engaged in rearing behavior.

### Analysis

Data for each behavioral element were grouped according to treatment and were initially checked for normality and homogeneity of variance. Levene's tests were statistically significant (i.e. assumptions that distributions were normal or variances were equal had to be rejected); therefore, non-parametric tests were conducted (as opposed to data transformation). Data were analyzed using Kruskal-Wallis nonparametric one-way analysis of variance across treatment groups. Where significant variations in the data were identified, post-hoc Mann-Whitney *U* comparisons with control condition were performed. In all cases, a standard significance level α was set at *p* = 0.05.

## Figures and Tables

**Table 1:** Correlations between study variables and descriptive statistics for combined experiments

	1	2	3	4	5	6	*M*	*SD*
1. Time Open							48.5	32.7
2. Open Entries	0.86***						5.5	3.7
3. Stretch Attend Posture	−0.66***	−0.52**					6.2	3.6
4. Head Dips	0.82***	0.77***	−0.68***				7.8	3.5
5. Risk Assessment	−0.81***	−0.68***	0.80***	−0.73***			25.4	21.3
6. Rearing Duration	0.28	0.29	−0.08	0.26	−0.25		11.5	4.0
7. Rearing Frequency	0.55**	0.54**	−0.29	0.37*	−0.47**	0.69***	7.2	2.1

*Notes.* * *p* < 0.05, ** *p* < 0.01, *** *p* < 0.001.

**Table 2:** Results from Experiment One

Diazepam					
*Behavior*	*Saline Control*	*0.25 mg/kg*	*0.5 mg/kg*	*0.75 mg/kg*	*H-values*
Time Open	27.5 (0–52.8)	55.8 (17.6–74.5)	53.2 (20.4–90.5)	50.9 (35.7–82.5)	5.077
Open Entries	4.0 (0–6.8)	5.0 (1.0–8.5)	3.5 (1.0–5.5)	8.5 (5.5–11.3)	7.797
SAP	11.5 (7.8–14.5)	5.5 (2.8–7.3)***	6.5 (4.8–9.3)**	4.0 (2.8–6.3)***	17.414***
Head Dips	5.5 (3.8–7.0)	7.0 (4.0–11.0)	10.5 (5.3–13.3)*	12.5 (11.0–15.8)**	15.648**
Risk Assessment	50.7 (32.6–67.2)	17.4 (6.4–33.8)**	20.9 (11.9–30.1)*	12.6 (5.9–26.3)**	11.744**
Rearing Duration	13.8 (11.6–23.7)	11.3 (6.0–28.2)	10.3 (4.0–19.4)	10.9 (6.3–17.1)	2.405
Rearing Frequency	8.0 (7.0–8.3)	8.0 (3.0–10.5)	6.5 (3.0–9.5)	6.0 (4.0–7.3)	3.060

RS 102221					
*Behavior*	*Saline Control*	*0.5 mg/kg*	*1.0 mg/kg*	*2.0 mg/kg*	*H-values*
Time Open	27.5 (0–52.8)	0.5 (0–52.8)	35.3 (0–78.0)	57.6 (30.9–92.6)*	8.550*
Open Entries	4.0 (0–6.8)	0 (0–1.0)	2.5 (0–10.3)	6.0 (1.8–9.3)	8.235*
SAP	11.5 (7.8–14.5)	3.5 (1.5–6.3)**	3.0 (1.5–4.3)***	4.0 (1.8–5.3)***	18.638***
Head Dips	5.5 (3.8–7.0)	3.5 (1.5–6.3)	7.0 (3.0–14.8)	14.5 (9.0–17.5)**	14.001**
Risk Assessment	50.7 (32.6–67.2)	46.8 (29.0–54.2)	10.3 (6.5–41.1)*	16.5 (4.5–30.9)**	12.495**
Rearing Duration	13.8 (11.6–23.7)	8.5 (1.7–15.1)	14.1 (6.6–19.8)	11.5 (6.0–20.1)	5.551
Rearing Frequency	8.0 (7.0–8.3)	5.5 (1.5–8.0)	6.5 (4.0–10.0)	6.5 (4.8–9.5)	5.865

MK-212					
*Behavior*	*Saline Control*	*0.25 mg/kg*	*0.5 mg/kg*	*0.75 mg/kg*	*H-values*
Time Open	27.5 (0.0–52.8)	4.9 (0.0–58.1)	9.0 (0.0–28.3)	0.0 (0.0–35.0)	1.584
Open Entries	4.0 (0.0–6.8)	1.0 (0.0–4.0)	0.5 (0.5–3.0)	0.0 (0.0–1.0)	5.105
SAP	11.5 (7.8–14.5)	12.5 (10.0–18.8)	14.0 (5.2–18.2)	8.0 (5.5–9.5)	7.271
Head Dips	5.5 (3.8–7.0)	6.0 (3.5–7.2)	2.5 (0.0–6.0)	3.0 (1.5–4.8)	5.821
Risk Assessment	50.6 (32.6–67.2)	81.6 (45.8–112.0)	51.4 (35.9–75.8)	53.5 (33.9–63.7)	4.873
Rearing Duration	13.8 (11.6–23.7)	10.1 (5.2–13.9)*	8.8 (4.7–16.0)*	6.1 (1.1–10.2)*	12.684**
Rearing Frequency	8.0 (7.0–8.2)	4.5 (2.5–6.5)**	5.5 (0.5–3.0)	3.0 (0.8–6.0)**	13.973**

*Notes.* * < 0.05, ** < 0.01, *** < 0.001, SAP = Stretched Attend Posture. Data expressed as medians (lower to upper quartiles), *H*-values based on Kruskal-Wallis, *p*-values based on Mann-Whitney *U* post-hoc comparisons with the control condition. Each dose condition included 10 animals with a total of 100 animals included in the study.

**Table 3:** Results from Experiment Two

Diazepam					
*Behavior*	*Saline Control*	*0.25 mg/kg*	*0.5 mg/kg*	*0.75 mg/kg*	*H-values*
Time Open	34.1 (5.0–83.0)	11.0 (0.0–26.3)	80.8 (21.0–114.7)	61.8 (13.5–87.6)	8.679*
Open Entries	4.0 (0.8–8.5)	1.5 (0.0–5.0)	9.5 (1.5–13.2)	6.5 (2.8–10.2)	4.835
SAP	8.5 (4.0–11.2)	7.5 (5.0–10.0)	4.0 (1.8–5.2)*	2.0 (0.8–5.0)*	13.890**
Head Dips	5.5 (2.8–8.2)	3.5 (2.0–5.2)	12.5 (6.5–17.0)*	8.5 (6.0–10.5)	14.109**
Risk Assessment	26.4 (11.0–42.0)	55.2 (36.0–63.6)	10.6 (0.0–17.9)	4.0 (0.0–17.0)**	17.218***
Rearing Duration	11.8 (6.4–27.3)	6.6 (2.9–19.2)	12.8 (8.8–19.9)	6.8 (2.4–18.4)	2.483
Rearing Frequency	7.0 (4.0–8.2)	5.0 (1.0–8.0)	6.5 (4.8–11.0)	5.0 (2.0–13.2)	2.490

Y-25130					
*Behavior*	*Saline Control*	*0.1 mg/kg*	*1.0 mg/kg*	*3.0 mg/kg*	*H-values*
Time Open	34.1 (5.0–83.0)	18.4 (18.2–28.5)	97.5 (83.2–32.8)*	43.0 (16.5–70.6)	13.267**
Open Entries	4.0 (0.8–8.5)	4.0 (2.8–7.2)	7.0 (5.0–12.0)*	7.0 (1.5–9.8)	16.487**
SAP	8.5 (4.0–11.2)	9.5 (8.5–11.0)	4.0 (3.0–5.0)*	7.5 (4.0–11.2)	12.880**
Head Dips	5.5 (2.8–8.2)	4.0 (2.8–7.2)	11.0 (9.0–12.2)*	9.5 (1.0–13.2)	8.745*
Risk Assessment	11.8 (6.4–27.3)	44.6 (24.8–66.0)	8.0 (0.0–12.9)**	14.7 (6.7–29.0)	16.584***
Rearing Duration	26.4 (11.0–42.0)	12.9 (12.2–14.2)	15.0 (11.4–18.2)	19.8 (15.3–23.3)	3.760
Rearing Frequency	7.0 (4.0–8.2)	9.5 (6.8–10.2)	9.0 (7.8–11.0)	8.5 (7.0–12.0)	6.352

SR 57227A					
*Behavior*	*Saline Control*	*0.1 mg/kg*	*1.0 mg/kg*	*3.0 mg/kg*	*H-values*
Time Open	34.1 (5.0–83.0)	65.0 (27.6–101.9)	35.7 (21.4–70.3)	2.3 (0.0–51.8)	8.477*
Open Entries	4.0 (0.8–8.5)	9.0 (3.2–13.5)	8.0 (2.8–9.5)	1.0 (0.0–12.2)	5.668
SAP	8.5 (4.0–11.2)	8.5 (5.5–14.2)	13.0 (9.5–17.2)	10.0 (5.8–14.2)	5.943
Head Dips	5.5 (2.8–8.2)	6.5 (6.0–14.5)	5.0 (2.0–6.0)	2.5 (1.0–4.8)	11.106*
Risk Assessment	11.8 (6.4–27.3)	24.6 (5.4–56.7)	53.2 (44.1–77.8)**	20.8 (15.7–46.2)	9.075*
Rearing Duration	26.4 (11.0–42.0)	12.2 (7.0–18.7)	7.4 (5.0–9.6)	7.1 (3.9–10.3)	5.808
Rearing Frequency	7.0 (4.0–8.2)	7.5 (6.8–9.2)	5.0 (3.8–6.2)	5.5 (3.5–7.0)	8.114*

*Notes.* * < 0.05, ** < 0.01, *** < 0.001, SAP = Stretched Attend Posture. Data expressed as medians (lower to upper quartiles), *H*-values based on Kruskal-Wallis, *p*-values based on Mann-Whitney *U* post-hoc comparisons with the control condition. Each dose condition included 10 animals with a total of 100 animals included in the study.

**Table 4:** Results from Experiment Three

Diazepam					
*Behavior*	*Saline Control*	*0.25 mg/kg*	*0.5 mg/kg*	*0.75 mg/kg*	*H-values*
Time Open	13.5 (0.0–40.8)	39.7 (20.9–53.6)	68.6 (50.1–120.7)**	75.8 (28.6–84.4)**	14.93**
Open Entries	1.0 (0.0–4.2)	4.5 (2.0–7.2)	12.5 (6.8–13.2)***	6.5 (1.8–9.0)*	14.44**
SAP	8.0 (1.0–11.2)	5.0 (3.0–10.0)	2.0 (0.8–4.0)	1.0 (0.0–3.8)*	9.01*
Head Dips	3.0 (2.8–5.8)	9.0 (3.0–12.2)	11.0 (7.0–15.8)**	10.5 (4.8–12.5)*	11.08*
Risk Assessment	52.2 (33.8–83.6)	22.6 (13.6–53.8)	8.8 (0.4–23.7) ***	4.6 (0.0–43.8)*	13.15**
Rearing Duration	15.6 (5.4–29.6)	16.2 (11.2–21.6)	11.2 (5.4–21.4)	4.8 (1.7–9.6)*	8.66*
Rearing Frequency	7.5 (4.0–9.2)	9.5 (7.2–11.0)	8.0 (4.8–10.2)	3.5 (1.8–5.5)	9.51*

RS 39604					
*Behavior*	*Saline Control*	*0.01 mg/kg*	*0.1 mg/kg*	*1.0 mg/kg*	*H-values*
Time Open	13.5 (0.0–40.8)	126.1 (103.8–163.7)**	88.8 (63.5–123.6)**	82.8 (57.8–109.9)**	23.19***
Open Entries	1.0 (0.0–4.2)	14.0 (12.8–17.5)***	7.0 (3.8–9.5)**	10.0 (7.8–12.5)***	26.52***
SAP	8.0 (1.0–11.2)	2.0 (0.0–5.2)	4.0 (3.8–5.0)	4.0 (2.8–6.2)	7.42
Head Dips	3.0 (2.8–5.8)	14.5 (11.0–16.5)***	8.5 (6.8–10.2)**	9.5 (6.0–10.2)**	18.76***
Risk Assessment	52.2 (33.8–83.6)	2.0 (0.0–9.7)**	4.3 (0.0–11.3)**	5.9 (2.3–15.5)**	19.47***
Rearing Duration	15.6 (5.4–29.6)	12.8 (9.2–23.2)	18.7 (8.3–24.2)	18.8 (11.7–22.5)	0.56
Rearing Frequency	7.5 (4.0–9.2)	10.0 (5.5–15.5)	9.0 (7.0–11.2)	11.0 (9.5–14.2)	6.61

RS 67333					
*Behavior*	*Saline Control*	*0.01 mg/kg*	*0.1 mg/kg*	*0.5 mg/kg*	*H-values*
Time Open	13.5 (0.0–40.8)	79.8 (41.0–103.2)**	82.2 (40.0–100.6)**	53.6 (21.0–83.8)	12.58**
Open Entries	1.0 (0.0–4.2)	10.0 (7.8–14.5)**	6.5 (2.0–10.2)*	5.5 (3.2–9.5)*	13.11**
SAP	8.0 (1.0–11.2)	2.0 (1.8–3.2)	3.5 (2.8–4.2)	8.0 (3.8–10.5)	8.88
Head Dips	3.0 (2.8–5.8)	10.5 (9.2–12.5)**	9.0 (7.8–11.2)**	8.0 (6.0–11.0)	12.48**
Risk Assessment	52.2 (33.8–83.6)	0.4 (0.0–0.6)*	9.2 (3.3–15.1)*	27.2 (13.7–38.1)*	23.57***
Rearing Duration	15.6 (5.4–29.6)	12.4 (5.4–17.2)	5.0 (2.9–13.6)	12.4 (8.8–17.3)	5.43
Rearing Frequency	7.5 (4.0–9.2)	12.0 (6.0–15.0)	8.5 (5.2–11.2)	7.5 (5.8–9.8)	2.66

*Notes.* * < 0.05, ** < 0.01, *** < 0.001, SAP = Stretched Attend Posture. Data expressed as medians (lower to upper quartiles), *H*-values based on Kruskal-Wallis, *p*-values based on Mann-Whitney *U* post-hoc comparisons with the control condition. Each dose condition included 10 animals with a total of 100 animals included in the study.

**Table 5:** Summary of significant findings across three experimental studies relative to saline controls

Drug	Dose	Open Arms		SAP	Head	Risk	Rearing	
	*(mg/kg)*	*Time*	*Entries*		Dips	Assessment	*Duration*	*Frequency*
**Experiment One**								
Diazepam	0.25	-	-	**↓**	-	**↓**	-	-
Diazepam	0.50	-	-	**↓**	**↑**	**↓**	-	-
Diazepam	0.75	-	-	**↓**	**↑**	**↓**	-	-
RS 102221	0.50	-	-	**↓**	-	-	-	-
RS 102221	1.0	-	-	**↓**	-	**↓**	-	-
RS 102221	2.0	**↑**	-	**↓**	**↑**	**↓**	-	-
MK-212	0.25	-	-	-	-	-	**↓**	**↓**
MK-212	0.50	-	-	-	-	-	**↓**	-
MK-212	0.75	-	-	-	-	-	**↓**	**↓**
**Experiment Two**								
Diazepam	0.25	-	-	-	-	-	-	-
Diazepam	0.50	-	-	**↓**	**↑**	-	-	-
Diazepam	0.75	-	-	**↓**	-	**↓**	-	-
Y-25130	0.1	-	-	-	-	-	-	-
Y-25130	1.0	**↑**	**↑**	**↓**	**↑**	**↓**	-	-
Y-25130	3.0	-	-	-	-	-	-	-
SR57227A	0.1	-	-	-	-	-	-	-
SR57227A	1.0	-	-	-	-	**↑**	-	-
SR57227A	3.0	-	-	-	-	-	-	-
**Experiment Three**								
Diazepam	0.25	-	-	-	-	-	-	-
Diazepam	0.50	**↑**	-	-	**↑**	**↓**	-	-
Diazepam	0.75	**↑**	-	**↓**	**↑**	**↓**	**↓**	-
RS 39604	0.01	**↑**	**↑**	-	**↑**	**↓**	-	-
RS 39604	0.1	**↑**	**↑**	-	**↑**	**↓**	-	-
RS 39604	1.0	**↑**	**↑**	-	**↑**	**↓**	-	-
RS 67333	0.01	**↑**	**↑**	-	**↑**	**↓**	-	-
RS 67333	0.1	**↑**	**↑**	-	**↑**	**↓**	-	-
RS 67333	0.5	-	**↑**	-	-	**↓**	-	-

*Notes.* SAP = Stretched Attend Posture, ↑ = increased behaviour (compared to saline control) as detected by Mann-Whitney *U* when Kruskal Wallis *H*-value < 0.05, ↓ = decreased behaviour (compared to saline control) as detected by Mann-Whitney *U* when Kruskal Wallis *H*-value < 0.05.
